# Health Sector Resource Mapping in Malawi: Sharing the Collection and Use of Budget Data for Evidence-Based Decision Making

**DOI:** 10.9745/GHSP-D-21-00232

**Published:** 2021-12-31

**Authors:** Ian Yoon, Pakwanja Twea, Stephanie Heung, Sakshi Mohan, Nikhil Mandalia, Saadiya Razzaq, Leslie Berman, Eoghan Brady, Andrews Gunda, Gerald Manthalu

**Affiliations:** a Clinton Health Access Initiative, Malawi.; b Ministry of Health, Government of Malawi, Malawi.; c Clinton Health Access Initiative, South Africa.

## Abstract

**Background:**

In 2011, the Ministry of Health in Malawi developed and institutionalized a resource-tracking process, known as resource mapping (RM), to collect information on planned funding flows across the health sector to support resource allocation and mobilization decisions. We analyze the RM process and tools and describe key uses of the data for health financing decision making to achieve universal health coverage (UHC).

**Methods:**

We applied a case study approach, written as a collaboration between policy makers who have led the RM process in Malawi and the implementation team who have developed tools, collected data, and reported results over the period. It draws on our experiences in conducting RM in Malawi to document the RM process and data, key uses of data, implementation challenges, and lessons learned. We conducted a gray literature review to understand rounds of RM in which we did not participate. Finally, we conducted a search of published literature to situate our work in the international health resource-tracking literature.

**Results:**

The RM exercise in Malawi is iteratively designed around the needs of the end users and policy priorities of the government, which in turn drives institutionalization of the exercise. We describe 4 ways in which RM data has been used, including national and district planning and budgeting; prioritization and coordination of existing funds by estimating resource availability; mobilization of new resources by conducting financial gap analysis against costed national strategic plans; and generation of evidence to support the national response to the coronavirus disease 2019 pandemic.

**Discussion:**

To achieve UHC goals in Malawi, RM has equipped the government and development partners with critical data used for resource mobilization and coordination decisions. Lessons learned from RM in Malawi may be applicable to other countries starting or refining their own health resource-tracking exercise.

## Abstract (precis)

By tracking budgets for health through its annual resource mapping exercise, the Government of Malawi generated evidence for planning and budgeting, quantifying resource needs, mobilizing funds to fill financial gaps, and coordinating investments across stakeholders with different priorities toward common goals. The exercise was adapted to conduct COVID-19 resource mapping to inform planning and coordination of the national pandemic response.

## Teaser

**Key Findings:**

- Resource mapping, an exercise conducted by the Government of Malawi, has tracked budgets for health since 2011 using a census-survey data collection approach.
- We describe 4 ways that data have been used to improve efficient allocation of resources and mobilize new funding for government priorities. This includes national- and district-level planning and budgeting, prioritizing and coordinating existing funds by estimating resource availability, mobilizing new resources by conducting financial gap analyses against costed national strategic plans, and generating evidence to support the national response to the coronavirus disease 2019 (COVID-19) pandemic.
- Institutionalization of the resource mapping process and tools has been supported by iterative design improvements that mitigate implementation challenges and ensure that collected data are relevant to the needs of policy makers. In turn, this drives demand for resource mapping data from health sector stakeholders.

**Key Implications:**

- Policy makers should ensure that health resource-tracking exercises are fit for their purpose and designed around the end user.
- A flexible approach to resource tracking is advantageous if resource allocation decisions are to be responsive to health emergencies such as the COVID-19 pandemic.

## INTRODUCTION

The Government of Malawi (GOM) strives to “provide adequate health care, commensurate with the health needs of Malawian society and international standards of health care.” As articulated in the National Health Policy 2017–2030 and the Health Sector Strategic Plan II 2017–2022, this includes strengthening the health system and addressing social determinants of health.^1^^,^^2^ Moreover, the Ministry of Health (MOH) has committed to providing the Essential Health Package of health services free to all Malawians. This is an important policy commitment toward the achievement of the Sustainable Development Goals and universal health coverage (UHC) to increase the inclusivity, availability, and equity of health services.^3^ GOM financially supports UHC, spending 10% of general government expenditure on health per annum, 2 percentage points higher than the average low-income country.^4^ Despite this commitment, evidence suggests significant gaps to UHC coverage remain.^5^^,^^6^

Malawi's limited economic capacity has restricted GOM health expenditure to US$9.6 per capita in 2017,^7^ which falls short of the World Health Organization (WHO) recommendation of US$86 per capita per annum needed for UHC.^8^ Thus, financial support from development partners has been critical for the health sector in Malawi. According to WHO, partner funding from 2009 until 2017 was approximately US$27 per capita per annum, or 63% of total spending on health compared with a regional average of 27%.^9^ These funding flows are fragmented and complex, with an estimated 115 financing sources and 225 implementing agents operating in Malawi's health sector.^10^

Resolving 2 key health financing challenges could improve UHC quality and coverage. First, funding fragmentation hampers efficient allocation of resources, since MOH must coordinate the numerous funding sources and implementing partners toward its priorities.^11^ Second, low absolute spending on health limits availability and access to the Essential Health Package. MOH must mobilize resources for health despite economic constraints on government income generation.

To promote efficient resource allocation and mobilization, the MOH Department of Planning and Policy Development established an annual resource mapping (RM) exercise in 2011 with technical support from the Clinton Health Access Initiative (CHAI).^12^ The exercise, similar to those of other countries in Africa including the Democratic Republic of Congo, Liberia, Senegal, Somalia, and Zambia, seeks to improve the availability of health financing data for decision making by collecting and consolidating standardized budget information from all health sector funders and implementers, including government and development partners, into a single database.^13^ Aggregating and analyzing health financing data increases the transparency of funding and supports MOH and partners in coordinating activities and mobilizing resources toward national priorities.

The resource mapping exercise seeks to improve the availability of health financing data for decision making by consolidating budget information from all health sector funders and implementers into one database.

The objective of this case study is to document policy makers' perspectives on the development and implementation of RM in Malawi. In addition, we show how RM data have been used to inform evidence-based planning, budgeting, and other decision making to improve health sector outcomes toward UHC. Finally, we highlight the availability of RM data for policy makers and development partners in Malawi as well as the challenges and lessons learned in conducting RM for countries developing or refining their own RM exercises.

## METHODS

Our analysis applies a case study approach, written as a collaboration between policy makers who have led the RM process in Malawi and the implementation team who have developed tools, collected data, and reported results over the period. It draws on our experiences in participating and leading RM in Malawi to document the RM process and data, key uses of data, implementation challenges, and lessons learned.

We supplement the experiences of both MOH and CHAI with an analysis of the RM gray literature informed by expert knowledge. This process was critical for understanding the rounds of the exercise in which we did not participate. We reviewed RM data collection tools, financial commitment datasets for the 6 rounds of the exercise to understand changes made to the data collection and analysis tools and processes. GOM program documents, funding applications, and partner documentation on lessons learned provided additional examples of how RM data had been used and identified successes and challenges beyond our own experiences. Table 1 provides a summary of the documents reviewed.

**TABLE 1.** Gray Literature Documents on Resource Mapping in Malawi Included in Review

	Government Program Documents	Partner Program Documents	Funding Requests	Resource Mapping Databases	Resource Mapping Data Collection Tools
Number of documents	57	49	3	6	6

Finally, we contextualized the authors' experiences and the gray literature review within the international resource-tracking literature through a search of English language studies published from 2011 to 2020 using PubMed, Web of Science, and JSTOR. Our search terms were the following: resource mapping, resource tracking, expenditure tracking, expenditure review, health budget tracking, health resource mapping, health resource tracking, health expenditure tracking, health expenditure review, and health accounts. We retrieved 271 published studies and found that while some studies related to national resource tracking, they tended to focus on single programs or disease areas (20) or expenditure estimates through national health accounts (135). Our search revealed a paucity of published research specific to resource tracking for health in Malawi and the region. We found only 1 such published study analyzing health expenditures^14^ and 1 Government of Malawi unpublished internal data document. Neither referenced the MOH-led RM exercise, which was ongoing at the time of these studies' publication.

## RESOURCE MAPPING: PROCESS AND DATA

From 2011 to the time of writing, 6 rounds of RM have been conducted in Malawi with the seventh round in progress to answer the following key questions:

- What is the total committed funding for health, and how does it vary over time?
- Who is planning to fund and implement health programs?
- How is the funding distributed across disease areas, interventions, geographical areas, and cost inputs?
- How is funding aligned to government priorities per its national strategic plans?

The data are collected through a customized classification system that captures the necessary parameters (Table 4) for each financial commitment and is embedded in a data collection template (Supplement). Table 2 illustrates the scope and comprehensiveness of RM. MOH has tracked approximately US$15 billion of financial resources for health from 2011 to 2020, with an average of 177 submitting organizations per year (unpublished internal data, Government of Malawi). These organizations include government ministries, departments, and agencies; bilateral/multilateral partners; and nongovernmental organizations. Private funding, such as out-of-pocket payments and voluntary health insurance, is not tracked through RM.

**TABLE 2.** Summary of Data Collected During 6 Rounds of the Ministry of Health Resource Mapping Exercises in Malawi

	Round 1 2011–2012	Round 2 2012–2013	Round 3 2013–2014	Round 4 2014–2015	Round 5 2017–2018	Round 6 2019–2020
Submitting organizations	33	233	229	165	235	166
Financing sources	54	219	216	176	201	185
Implementing agents	163	290	221	262	274	226
No. of years of data collected	5(2011–2016)	4(2012–2016)	6(2012–2018)	5(2014–2019)	3(2017–2020)	5(2015–2020)
Total funds captured	US$ 2.40 Billion	US$ 2.48 Billion	US$ 2.79 Billion	US$ 2.29 Billion	US$ 1.80 Billion	US$ 3.09 Billion
Type of financial data collected	Budget only	Budget only	Budget and exp.	Budget only	Budget only	Budget and exp.

**FIGURE 1:**
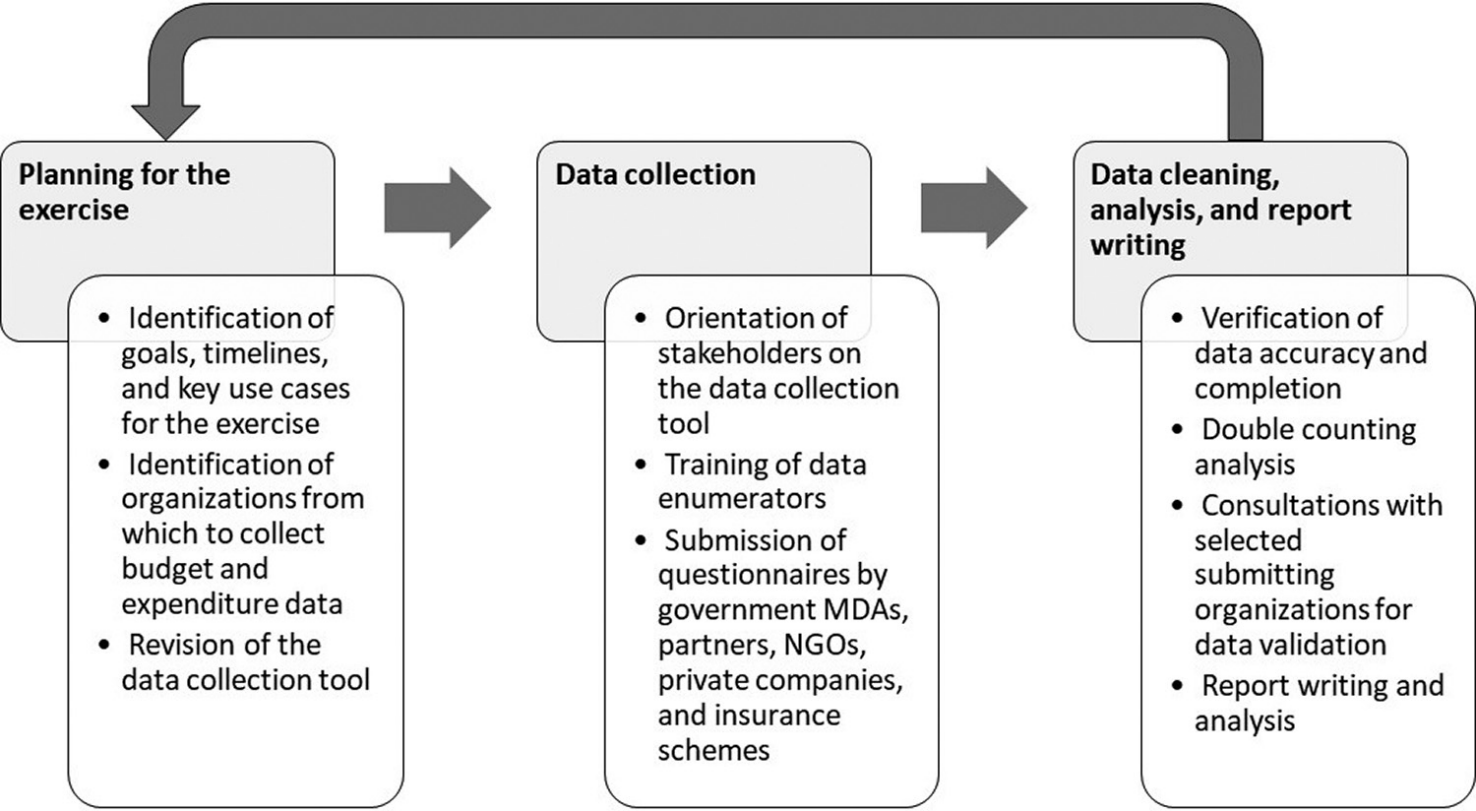
Example Resource Mapping Process Schematic Abbreviations: MDAs, ministries, departments, and agencies; NGOs, nongovernmental organizations.

As shown in Figure 1, the RM process is divided into 3 stages: (1) planning and updating of data collection tools; (2) data collection, cleaning, and consolidation; and (3) data analysis, results dissemination for use in planning, and solicitation of feedback. Importantly, the RM process is cyclical, with learnings from previous rounds feeding into the planning and implementation of future exercises.^10^ The RM exercise is iteratively designed so that it remains relevant to policy decisions and straightforward to conduct.

## USES OF RESOURCE MAPPING DATA IN DECISION MAKING

The iteratively designed process and data collection tool has enabled the generation of RM data that can inform a range of health financing decisions. We classify the use cases of RM data into 4 categories: (1) planning and budgeting, (2) improving allocative efficiency through coordination of existing funding, (3) securing increased funding for quantified resource gaps, and (4) responding to the coronavirus disease (COVID-19) pandemic.

The iteratively designed process and data collection tool has enabled generation of RM data that can inform a range of health financing decisions.

### Planning and Budgeting

RM data are used by MOH during its annual budgeting process at both the national and district level. At the national level, RM data are imported into the annual MOH budgeting tool and provide MOH departments with visibility into existing development partner funding during the development of annual government workplans and budgets.

RM data are also used for district planning, budgeting, and partner coordination by GOM district health offices (DHOs). Integration of RM data into the district planning process supports DHOs in identifying earmarked commitments for their district. However, as data collection is conducted at the national level, district-level figures may not reflect actual budgets in the district. Therefore, as part of the government's routine planning and budgeting cycle, DHOs validate national RM estimates with a district-level census of commitments from development partners to fully understand the financial envelope for health in their specific district. By collecting more granular, accurate funding information, DHOs can enhance coordination and alignment between district governments and their partners (unpublished internal data, Government of Malawi).

### Improving Efficiency Through Coordination of Existing Funding

National strategic plans (NSPs) are instrumental in defining and coordinating a cohesive approach for governments and partners. With an estimated 115 funding sources and 227 implementing partners in the country, these plans are critical for minimizing both duplication and transaction costs and improving efficiency.^10^^,^^15^ RM data have spurred prioritization of NSP activities by illustrating the funding constraints for diseases, interventions, and health systems areas. For example, the HIV/AIDS NSP 2015–2020 estimated HIV resource availability using RM data to inform a participatory prioritization process. The initial cost of the HIV NSP 2015–2020 was US$1.7 billion, which was greater than the US$1.6 billion available. The resource need was driven by HIV testing costs with ambitious treatment targets requiring larger testing volumes. An aggregation of testing budgets using RM data showed insufficient funding compared with the testing cost estimated in the plan, suggesting that the proposed testing strategy was financially unrealistic. Using this information, the National AIDS Commission engaged civil society organizations, MOH, and development partners to optimize HIV testing strategies under different resource availability scenarios. As a result, these stakeholders agreed to a coordinated and targeted testing strategy with estimated savings of US$42 million over 5 years. Analyses were also conducted by activity type to demonstrate potential duplication in activities such as trainings. This evidence supported stakeholders in redirecting overlapping funding toward other underfunded, priority interventions. Thus, the HIV NSP 2015–2020 costs were brought in line with available funding, while also maintaining the plan's service delivery targets.^16^

### Secured Funding for Quantified Resource Gaps

Gap analyses for programs and activities using RM data and NSP costs can support the development of investment cases for resource mobilization. For instance, by conducting HIV and tuberculosis gap analyses with RM data, MOH has mobilized resources and informed programming of approximately US$1.3 billion through 3 successful grant applications to the Global Fund since 2012.^17^^–^^19^ The flexibility of the RM classification system and tool was critical because both MOH strategic priorities and the Global Fund requirements changed between 2012 and 2019. For example, in preparation for the 2015–2017 application, the RM classification system was modified so that funding for activities in the dataset could be disaggregated by beneficiary groups and programmatic interventions in accordance with Global Fund specifications. This mapping produced detailed estimates of resource availability against forecasted financial need, supporting MOH in articulating its investment case within the framework of the Global Fund funding mechanism.

### COVID-19 Resource Mapping

With the emergence of the COVID-19 pandemic in early 2020, GOM launched its National COVID-19 Response and Preparedness Plan in April 2020. This multisectoral plan aimed to prepare for a timely, coordinated pandemic response and to limit the spread of COVID-19 into the country.

To support resource mobilization, limit duplication, and monitor movement of financial commitments away from other essential health services, MOH initiated a rapid COVID-19 Resource Mapping exercise (COVID RM). This exercise built upon the pre-existing institutional capacity developed through the 6 rounds of the annual sector-wide RM. The government's familiarity with the existing RM process as well as the flexibility of the RM tool and classification system expediated modification of the RM exercise for COVID-19 resource tracking. With COVID RM, MOH tracks commitments against the objectives and activities of the National COVID-19 Plan. The tool was rolled out for data collection by partners who were accustomed to the sector-wide RM exercise and needed minimal assistance in submitting their COVID-19 budgets. As a result, data collection has been accelerated, enabling frequent updating of commitments and ensuring that data remain relevant and accurate.

**FIGURE 2:**
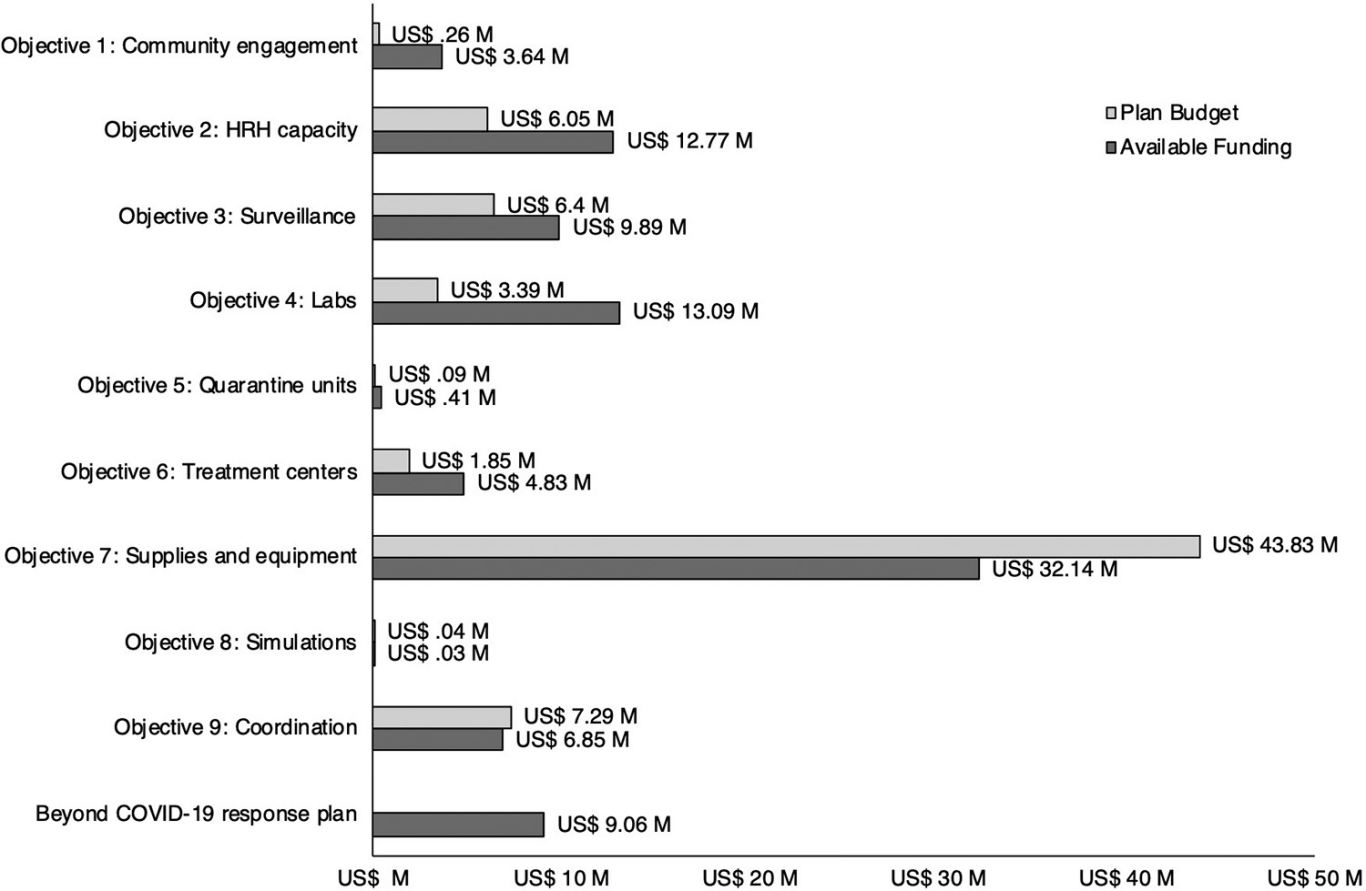
Funding Availability and Budgets for Malawi's COVID-19 National Response Plan (2020) Abbreviations: HRH, human resources for health; M, million.

COVID RM data can be used to inform resource allocation decisions at both the national and district level. For example, as Figure 2 shows, US$32.1 million is available for Objective 7: Supplies and Equipment. This amount is below the resource requirements outlined in the National COVID-19 Plan and suggests a funding gap of approximately US$11.7 million, thus justifying additional investment.

COVID RM data can also be disaggregated to the district level. This information has been shared with the DHOs to support subnational COVID-19 planning, resource allocation, and partner coordination. Partner commitments for COVID-19 are incorporated into the DHOs' annual district health plans. This process ensures that activity funding is transparent to all stakeholders and supports effective coordination of the COVID-19 response subnationally (unpublished internal data, Government of Malawi).

## DISCUSSION

### Challenges and Lessons Learned

Because the exercise is iteratively designed, each successive round of RM can incorporate lessons learned from the previous round as well as respond to emerging issues. To illustrate the advantages of iterative design, we note 4 lessons from 6 rounds of RM and the adjustments made to respond to the issues involved. These lessons learned may be informative for other countries interested in developing or strengthening their own resource-tracking exercises.

Each successive round of RM can incorporate lessons learned from the previous round as well as respond to emerging issues.

1. Flexibility of the RM classification system must be leveraged so that the dataset can be rapidly adapted to answer MOH's priority policy questions. For instance, the COVID-19 pandemic presented a significant challenge as an entirely new set of programmatic classifications was required to estimate resource availability for novel interventions included in the national COVID-19 response. The remainder of the RM classification system remained intact, and thus only targeted updates to the data collection tool were needed (unpublished internal data, Government of Malawi).^10^ This approach was also supported by clear documentation of the classification adjustment process, including standard operating procedures and guidelines, that facilitated rapid adjustments to the RM tool. Flexible tool design and proactive identification of data end-users can enable resource-tracking exercises globally to better support evidence-driven resource allocation and coordination toward changing government priorities.
2. Through iterative learning, opportunities have been identified to expedite the RM process and ensure that data are available in a timely manner to inform GOM and partner decision making. Since a primary use of the data is to inform MOH planning and budgeting for the next financial year, completion of RM is time dependent. One challenge to timely completion of the exercise is the capacity of submitting organizations to complete the data entry tool. In the first round of RM, MOH headquarters and national-level partners participated in data entry trainings, which reduced the time and the number of follow-ups needed to complete the submission, as well as increased submission quality relative to DHOs and district-based implementing partners who did not receive these trainings. In subsequent rounds, these trainings were expanded to include DHOs as well as district-based implementing partners (unpublished internal data, Government of Malawi). Another improvement on the follow-up process was the prioritization of the largest financing organizations. RM shows that the 10 largest health funders provide approximately 90% of financial commitments to the health sector (unpublished internal data, Government of Malawi). Therefore, while all submitting organizations receive follow-up where needed, the RM team prioritizes close consultations and follow-ups with the 10 largest funders to ensure that the submissions from these organizations are of high quality. Focused prioritization can support resource-tracking teams globally in expediting completion of the exercise and navigating the trade-off between data quality versus timeliness.
3. Incorporating feedback mechanisms into the data collection tool to guide users through data entry can increase data quality. A challenge to data quality is that the diverse financial reporting systems of both GOM and development partners may not clearly map to the RM classification system, leading to erroneous data entry. To reduce these errors, automated data quality and completion checks were included in the tool to support organizations in reporting information as aligned to RM classifications. In addition, a dashboard providing a summary of total resources reported by the submitting organization was added so organizations could validate their budgets and address potential issues in advance of submission to MOH (unpublished internal data, Government of Malawi). Automated checks may assist resource-tracking exercises that have challenges with template completeness and data quality.
4. Impact of staff turnover can be reduced through development of standard operating procedures and checklists that codify data collection, cleaning, and analysis. Over 6 rounds of RM, average government turnover in the RM team between rounds was 70%. Such turnover slows the exercise and can reduce data quality as each new team must learn key steps in the RM process, and experiences and insights gained by previous team members may be lost. To improve exercise continuity despite turnover, an RM “toolkit” with these standard operating procedures and checklists was compiled to document learnings and support the induction of new team members (Table 3) (unpublished internal data, Government of Malawi). Clear and specific documentation can also support learning across resource-tracking exercises both within and across country contexts.

**TABLE 3.** Documents Compiled in Malawi Resource Mapping Toolkit to Support Institutionalization and Sustainability

Data Collection Planning	Database Consolidation and Cleaning	Analysis and Report Writing	Other Documents
Roadmap template	Database consolidation tool	RM report template	RM - NHA parameter mapping
Organization contact information database	Database consolidation SOP	RM analysis templates	
Submission tracker	Database standardization SOP		
Submission management protocol	Removal of double-counting SOP		
Data entry training materials			

Abbreviations: NHA, national health account; SOP, standard operating procedures.

**TABLE 4.** Data Parameters and Classification Used in 6 Rounds of Government of Malawi Resource Mapping

Type	Data Element	Definition of Data Element	Illustrative Example	Corresponding NHA Dimension
1. Financiers and Implementers	Submitting organization	Organization that submitted budgeting information	Action Aid	N/A
	Financing source	The organization or entity financing the activity	Global Fund	Health providers, health care financing schemes, revenues of health care financing schemes
	Primary implementing agent	Primary organization or entity that is carrying out implementation	Action Aid	
	Subimplementing agent	Additional organization or entity carrying out the activity as a subgrantee of the primary implementing agent, if applicable	Southern African AIDS Trust	
2. Programs, Projects, Activities	Project name	Specific project that is supported by the activity	TB/HIV epidemic control	N/A
	Activity	Free-form text to describe the specific activity within the intervention	Comprehensive programs for people in prisons	N/A
	Programmatic function	Programmatic area, function, or disease supported by the activity	HIV including viral hepatitis and other sexually transmitted infections	Health care function, disease classification
	Programmatic intervention level 1	General intervention supported by the activity, dependent on the programmatic function	Prevention	
	Programmatic intervention level 2	Detailed intervention supported by the activity, dependent on the programmatic intervention level 1	Behavior change communication for HIV	
	Essential Health Package intervention	Alignment to Malawi's Essential Health Package interventions	Community health promotion and engagement	N/A
	Target population	Subpopulation targeted for HIV, TB, and malaria interventions only	People in prisons and other closed settings	Beneficiary characteristics
3. HSSP II Alignment	HSSP II objective	Classification of activities according to the relevant HSSP II objectives	Human resources for health	Factors of provision
	HSSP II sub-area	Classification of activities according to the relevant HSSP II sub-areas, dependent on the selection for HSSP II objective	Health worker training, in-service	
4. Geography	District	Percentage of funding earmarked for specific district(s); if central level, can be specified as 100% central level	50% Mwanza, 50% Thyolo	N/A
5. In-Service Training Details	Type of training	Modality of the training, (e.g., on the job, offsite, or virtually/online)	Offsite	N/A
	Health worker cadre	The health worker cadre that the training focused on (e.g., pharmacy, nursing, laboratory, etc.)	Nursing	N/A
	Health worker cadre	The specific health worker cadre targeted by the training, dependent on the selection for health worker cadre	Nurse officer	N/A
	Number of health workers targeted	Total number of health workers trained from July 2019–June 2020	50	N/A
	Activity frequency	Frequency of the trainings (i.e., annually, biannually, quarterly, monthly, or other)	Quarterly	N/A
	Monthly implementation plan	Tentative implementation timeline for the training, disaggregated by month, from July 2019–June 2020	Implemented from March–May 2020	N/A
6. Currency and Budgeting	Currency	Currency of the submitting organization's budget	US$	Expenditure information
	Fiscal year start month	Fiscal year start month of the submitting organization	July	
	Expenditures by year	Expenditure amount per year for FY 2015/2016, FY 2016/2017, and FY 2017/2018	US$100,000 annually	
	Budgets by year	Budget amount per year for FY 2018/2019 and FY 2019/2020	US$150,000 annually	N/A

Abbreviations: FY, fiscal year; HSSP, Health Sector Strategic Plan; N/A, not applicable; NHA, national health accounts.

### Future Directions for Resource Mapping

**FIGURE 3:**
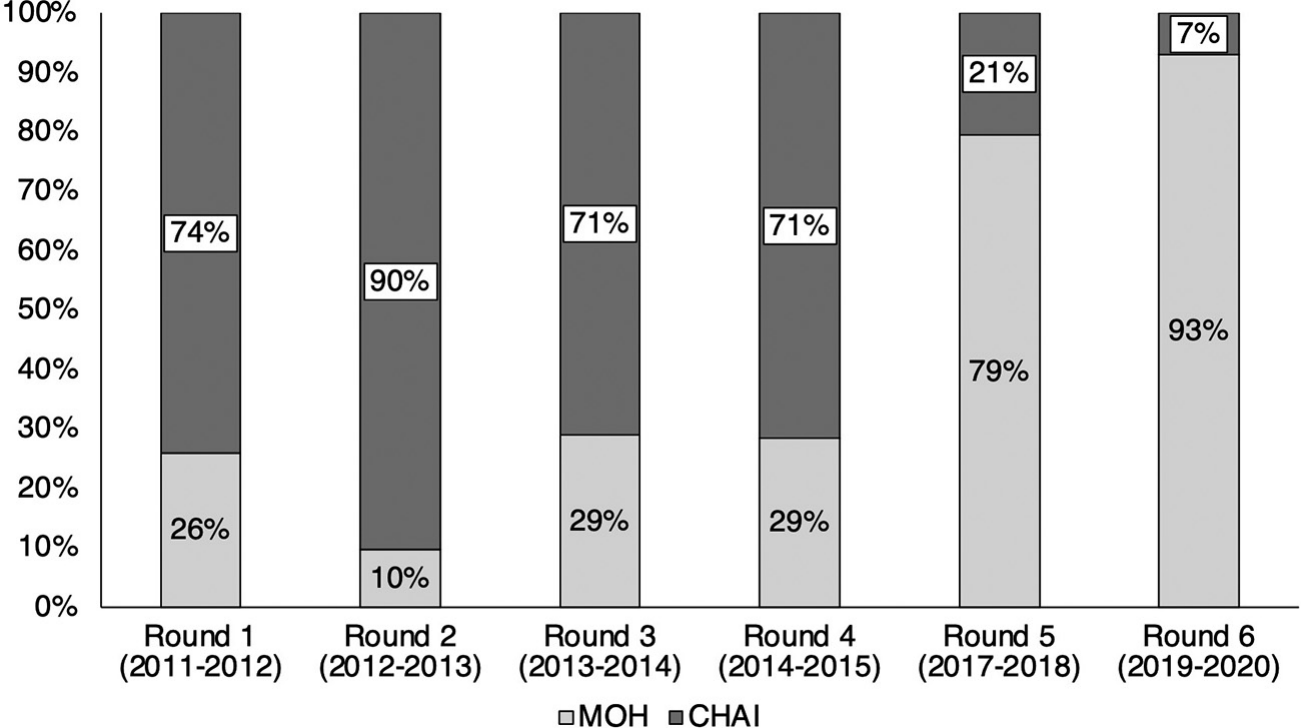
Human Resource Contribution for Resource Mapping in Malawi by Round (2011–2020) Abbreviations: CHAI, Clinton Health Access Initiative; MOH, Ministry of Health.

With 6 rounds completed, RM provides critical data used for resource mobilization and coordination and has been institutionalized within the annual MOH planning and budgeting processes. Figure 3 shows considerable, although nonlinear, progress has been made in shifting human resource contributions for RM away from CHAI and toward MOH. While MOH has always led the process, their human resource contribution initially focused on exercise planning, identifying use cases for the data, and disseminating results. Over time, MOH has increasingly invested human resources into other components of the exercise, including collecting and analyzing data, as well as mobilizing and managing funding for the exercise. External support now targets technical issues such as major tool modifications, classification system revisions, and ad hoc data analytics.

RM provides critical data used for resource mobilization and coordination and has been institutionalized within the annual MOH planning and budgeting processes.

Moving forward, the use of RM could be further enhanced in several ways. First, MOH can continue to encourage other health resource-tracking exercises to leverage the comprehensiveness of RM data and limit parallel or duplicative resource-tracking processes. For example, MOH recently harmonized WHO's national health accounts (NHA) with RM to produce estimates of budgets (RM) and expenditures (NHA) for health through a single data collection process. This process lowers the human resource burden for both submitting organizations and the MOH team and ensures that the same dataset is used consistently across the health sector.

Second, RM data could be analyzed to answer new questions of policy importance. For example, resource availability estimates from RM might be compared with Essential Health Package costs at the district level to assess if financial commitments are adequately supporting the provision of the benefits package and progression toward UHC. Additionally, district data could be used to assess the funding landscape at the subnational level since health budgets and the number of development partners vary across districts. Such analysis could inform targeted aid coordination interventions accounting for the severity and type of financial fragmentation in each district. However, since nationally collected data can often misrepresent district-level commitments, improving subnational aid coordination will inevitably require further budget transparency between DHOs, implementing partners, and funders.

Third, the RM team can continue to strengthen cross-country learning on RM best practices, as the RM processes and tools are often applicable between countries. For example, the harmonization of RM and NHA in Malawi drew from lessons learned from the Zimbabwe experience, specifically leveraging the crosswalk process between the RM and System of Health Accounts classification systems in Zimbabwe. The lessons learned from Malawi have also proved cross-applicable to others. For example, the Government of Ethiopia refined its own resource-tracking exercise building on the Malawian experience. However, cross-country learning is not confined to the technical details. Countries can also assess the relevance of RM data use in Malawi to both inform development or refinement of their own RM exercise and identify opportunities for data use.

### Limitations of Resource Mapping

The trade-off between data quality and data granularity remains a challenge for RM. For example, RM data capture the programmatic area (e.g., HIV/AIDS) as well as interventions within each area (e.g., HIV testing) and subinterventions (e.g., HIV self-testing). The sixth round of the exercise included 10 classifications for program areas, 54 classifications for programmatic interventions, and 73 classifications for programmatic subinterventions.^9^ This detailed system provides the granularity needed by different end users and has informed program-specific resource-tracking exercises. However, the classification system simultaneously reduces data quality as organizations face challenges in disaggregating their budgets by the RM categories. To mitigate this problem, the classification systems of the first 5 rounds of RM included a “cross-cutting” category that was meant to encompass “funding for health systems or general administrative costs as well as … programs that fund multiple disease areas but could not be … disaggregated.”^17^ However, the result was that 41% of total resources were reported as “cross-cutting” in the initial 5 rounds, suggesting inappropriate tagging of programs as “cross-cutting” and underreporting of programmatic financing. In the sixth round of the exercise, this category was converted to “health system strengthening,” prompting organizations to use the category for systems-strengthening activities only and fully disaggregate their programmatic financing elsewhere in the tool.^16^

Expanding the uptake of RM requires more detailed data to inform specific policy decisions, but data quality and timeliness may be compromised when the data solicited are too granular and do not map well to the existing financial systems of partner organizations. The RM exercise must therefore balance the trade-off between the granular data needed to inform the policy priorities of the end users versus the development of a streamlined, user-friendly tool that facilitates completion of the RM process in a timely manner.

Finally, RM is a method of tracking financial commitments from many disparate resource pools and implemented by many agents. However, a commitment to UHC may require greater efficiency in resource use through increased financial pooling of health services, as well as increased domestic resource mobilization. Ultimately, a government-funded, pooled system would not require RM as it is currently implemented. However, RM continues to fill an important information gap in the interim.

## CONCLUSION

To achieve ambitious UHC goals in Malawi, it is imperative that additional funding is mobilized for health and the limited funding available is efficiently allocated toward GOM's priorities. To this end, RM data have equipped GOM and development partners with critical information regarding available funding in the health sector. RM data enabled the quantification of resource needs, informed resource mobilization efforts to fill funding gaps, and supported coordination of disparate investments across stakeholders with different priorities and planning processes toward common goals. Moreover, the exercise's flexibility and the longstanding familiarity of government and partners with RM has facilitated rapid adaptation of the exercise to track resources for the COVID-19 response. Finally, an institutionalized RM will continue to be iteratively designed to ensure it remains relevant to the government's evolving policy priorities.

## Supplementary Material

21-00232-Yoon-Supplement.xlsx

GHSP-D-21-00232-Yoon-Supplement.xlsx

## Notes

**Peer Reviewed**

**First published online:** October 22, 2021.
